# Maternal burnout and participation in professional activities

**DOI:** 10.1192/j.eurpsy.2026.11935

**Published:** 2026-07-31

**Authors:** E. Polonetskaya, E. Pervichko

**Affiliations:** Faculty of Psychology, Lomonosov Moscow State University, Moscow, Russian Federation

## Abstract

**Introduction:**

Many mothers find themselves torn between their children and work. However, both of these domains are simultaneously important for many modern women and, therefore, need to be balanced in some way. To what extent specific preferences such as a flexible schedule, shorter working hours and work-from-home arrangements may help working mothers is also currently a subject of public discussion.

**Objectives:**

Our research was aimed at studying the associations between the intensity of participation in professional activities and other work-related arrangements, and maternal burnout during the first years of motherhood.

**Methods:**

A maternal burnout questionnaire in Russian (the Maternal Burnout Assessment Tool, MBAT), being an adaptation of the Burnout Assessment Tool to the motherhood context, was used to assess burnout tendencies in mothers. The sample consisted of 792 Russian-speaking mothers with children up to seven years of age. Single- and multi-factor analysis of variance was used for data analysis.

**Results:**

Our study demonstrates that participation in professional activities and the intensity of such (full-time or part-time employment) does not have a significant influence, either positive or negative, on the symptoms of maternal burnout.

As to specific work-related arrangements, as shown below, the highest levels of burnout are associated with the mixed arrangement type, when a mother works partially from home and partially outside the home (at her employer’s premises), while working outside the home is associated with the lowest levels of burnout (p=0.015). Mean burnout scores are as follows: not working - 2.59, working from home - 2.61, mixed arrangement - 2.89, working outside of home - 2.47.

**Conclusions:**

While the fact of participation in professional activities was not itself shown to have significance in our study, work-related arrangements appear to be essential, and working outside home is associated with lower burnout levels compared to mixed arrangement. Further and more detailed research on extensive samples is advisable.

**Disclosure of Interest:**

None Declared

